# Tumeurs rares de l'ovaire: à propos d'une série de 11 cas de tumeurs non épithéliales malignes de l'ovaire

**DOI:** 10.11604/pamj.2015.20.174.3446

**Published:** 2015-02-24

**Authors:** Nisrine Mamouni, Hanane Saadi, Sanaa Erraghay, Chahrazade Bouchikhi, Abdelaziz Banani

**Affiliations:** 1 Service de Gynécologie et Obstétrique I, CHU Hassan II, Fès, Maroc

**Keywords:** Tumeurs, ovaire, dysgerminome pur, Tumors, ovary, pure dysgerminoma

## Abstract

Les tumeurs non épithéliales malignes de l'ovaire représentent environ 20% des cancers de l'ovaire. L'objectif de notre travail est de dresser les particularités diagnostiques cliniques et d'imagerie de ces tumeurs. Nous avons procédé à une étude rétrospective portant sur 11 cas de tumeurs non épithéliales de l'ovaire. Ces tumeurs ont été colligées au service de gynécologie et obstétrique I du CHU Hassan II de Fès sur une période de 4 ans, entre janvier 2009 et décembre 2012. Les tumeurs germinales représentant 54% (6 patientes) des cas alors que les tumeurs du cordon sexuel ont été représentées par 4 cas de tumeurs de granulosa de type adulte et nous avons colligés un cas de lymphome ovarien primitif. La symptomatologie clinique était dominée par la distension abdominale associée souvent à des douleurs abdominopelviennes chroniques. La taille tumorale moyenne était de 175 mm avec un aspect solido-kystique dans 54% des cas. Le dosage des marqueurs tumoraux (hormone chorionique gonadotrope, lactate déshydrogénase, CA 125, alpha-fœtoprotéine) a été réalisé chez toutes les patientes. La découverte d'une masse annexielle suspecte chez une jeune femme doit, outre une tumeur frontière ou un cancer épithélial de l'ovaire, évoquer une tumeur non épithéliale, a fortiori si cette masse est volumineuse, si elle est associée à des signes d'hyperestrogénie ou d'androgénie.

## Introduction

Les tumeurs non épithéliales malignes de l'ovaire représentent moins de 20% des cancers de l'ovaire chez l'adulte. En dehors des tumeurs hématologiques, on distingue essentiellement les tumeurs germinales, les tumeurs du stroma et les tumeurs des cordons sexuels. Leurs âges de survenue apparaissent comme leurs pronostics et leurs traitements beaucoup plus hétérogènes que pour les formes épithéliales. Si l’échographie pelvienne en impose pour une tumeur maligne, certains éléments négatifs, comme l'absence de projection papillaire, un épanchement péritonéal de faible abondance et une mobilisation aisée de la masse sont primordiaux. Cette discordance, associée aux signes cliniques et au bilan biologique, doit faire suspecter une tumeur non épithéliale.

## Méthodes

Il s'agit d'une étude retrospective, réalisée du 1^er^ Janvier 2009 au 31 Décembre 2012. Le principal critère d'inclusion a été le type histologique de la tumeur. Nous avons inclus tous les cas de tumeurs germinales malignes de l'ovaire, des tumeurs stromales malignes et celles du cordon sexuel, ainsi que toutes les autres tumeurs non épithéliales malignes primitives de l'ovaire. Notre recueil de données concernait: âge, la date du diagnostic histologique, les éléments cliniques, biologiques et d'imagerie.

## Résultats

Durant cette période, nous avons rescencé 62 patientes chez qui le diagnostic de tumeur ovarienne maligne a été posé, parmi lesquels 11 cas de tumeur non épithéliale ovarienne maligne primitive. La fréquence de cette pathologie dans notre série est de 17,7 pour cent. Pour le groupe des tumeurs germinales malignes, l’âge moyen de nos patientes a été de 22 ans (extrêmes 17-30 ans) avec une parité moyenne de 1 (0-2). L’âge moyen du groupe des tumeurs stromales et cordon sexuel malignes est de 48 ans (extrêmes 29-61 ans) avec une parité moyenne de 3,5(0-7) (Tableau 1). Les circonstances de découverte ont été dominées par la distension abdominale chez 75% des cas, des douleurs abdominopelviennes dans 6 cas (50%) et l'apparition de métrorragie chez deux de nos patientes. Une de nos patiente a consulté pour l'apparition d'une dyspnée concomitante avec une distension abdominale (un cas de tumeur de la granulosa adulte avec une pleurésie gauche réactionnelle) (Tableau 2). Dans l'optique de préciser le diagnostic préopératoire, nous avons été souvent amené à s'aider des marqueurs tumoraux qui ne sont pas toujours spécifiques. En préopératoire, le marqueur CA125 a été demandé pour toutes nos patientes revenant positif dans tous les cas avec un taux moyen de 398 UI/ml. Le dosage de l'alphafœtoproteine a été réalisé chez deux patientes revenant normal dans le cas de tumeur germinale mixte et nettement augmenté dans le cas de tératome immature (3000ng/ml). Le dosage de LDH (lactate déshydrogénase) a été réalisé dans deux cas revenant positif (le dysgerminome pur et le lymphome de Burkit) avec un taux de 2991 et 866 UI/l respectivement. Le dosage de la fraction beta de l'hormone chorionique gonadotrophique a été réalisé dans trois cas revenant positif; le cas de tumeur germinale mixte de choriocarcinome et de tératome immature avec un taux de 2750, 200 et 100 UI/ml respectivement. Le dosage du taux d'ihnibine, des androgènes et de l’œtradiolémie n'a pas été réalisé vu le manque de moyens.

**Tableau 1:** Répartition des tumeurs non epitheliales ovariennes selon l’âge des patientes

Type histologique	Age moyen (Année)	Extrêmes
Tumeur germinales	22	17-30
Tumeur stroma et cordon sexuel	48	29-61
Tumeurs autres: Lymphome ovarien primitif	17	-

**Tableau 2:** Circonstances de découverte

	Nombre de cas	Age moyen	ménopausées	nullipares	CDD
Tumeur stroma et cordon sexuel	4	48	2	2	Masse abdominoplevienne:2 Metrorragie: 2 Aménorrhée:0 Virilisme:0 DAP: 2 Dyspnée: 1
Tumeurs germinales	7	22	0	4	Masse abdominopelvienne:7 DAP:4 Metrorragie: 0

CCD: circonstance de découverte. DAP: douleurs abdominopelviennes

**Figure 1:**
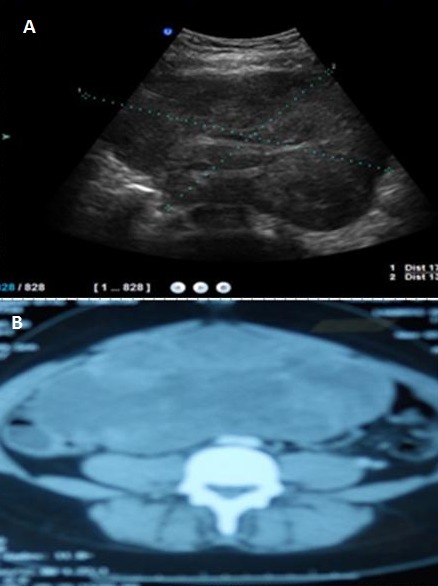
Dysgerminome pur: A/image echographique laterouterine hypoechogene sans epanchement peritoneal B/ une image tissulaire heterodense abdominopelvienne au scanner en coupe axiale

**Figure 2:**
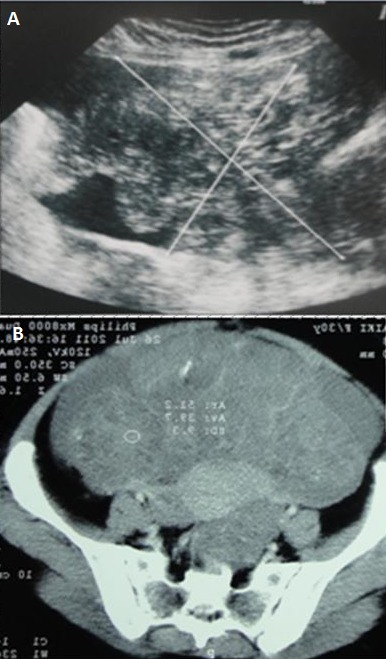
Teratome immature: A/image echogene hétérogène laterouterine avec épanchement péritonéal. TDM en coupe axiale: masse abdominopelvienne hétérodense avec des calcifications

**Figure 3:**
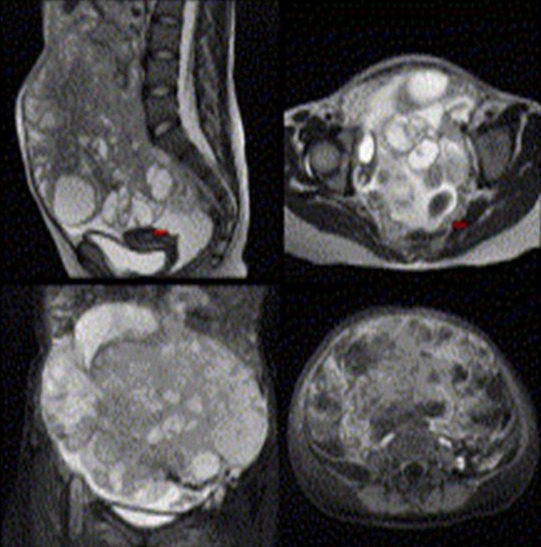
Tumeur germinale mixte: IRM abdominopelvienne: volumineuse masse lobulée bien limitée, en heterosignal en T2 avec multiples composantes kystique et tissulaire. L'utérus et l'ovaire droit sont visible (fléches rouges)

**Figure 4:**
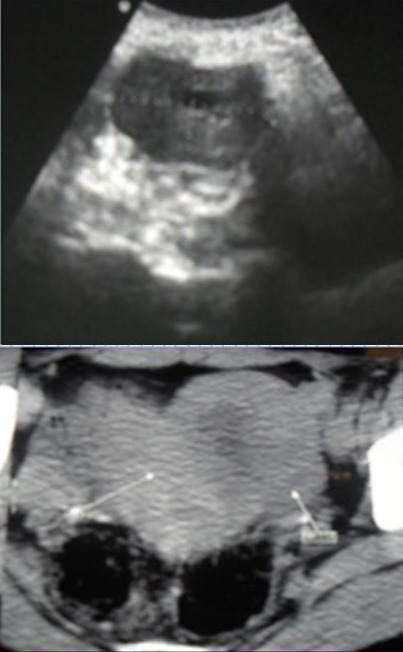
Tumeur de la granulosa: A/ une image echogéne tissulaire laterouterine. B/ TDM abdominopelvienne en coupe axiale: masse pelvienne heterodense latero-uterine

**Figure 5:**
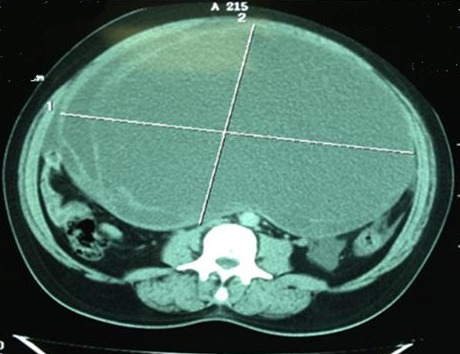
Tumeur de la granulosa adulte: TDM abdominopelvienne: volumineuse masse kystique abdominopelvienne avec des cloisons internes mesurant 40×35 cm

**Figure 6:**
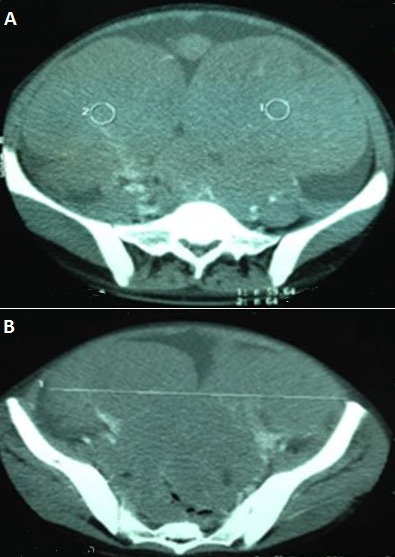
Lymphome de burkitt primitif de l'ovaire: TDM abdominopelvienne: deux volumineuses masses pelvienne tissulaires, droite: 15cm et gauche 18cm, hétérogenes avec des polyadenopathies iliaques bilaterales. Ascite abondante

Nous avons essayé de regrouper des critères échographiques pour mieux évaluer le risque de malignité des masses ovariennes ainsi que leur origine épithéliale ou non épithéliale. Toutes nos patientes ayant bénéficié au préalable d'une échographie pelvienne, par voie sus pubienne et endovaginale (chez les patientes non vierges) avec le recours à une cartographie Doppler couleur (Tableau 3). La sensibilité de l’échographie pour le diagnostic de bénignité ou malignité dans notre étude a été forte de 90,42%. La tumeur a été solido-kystique dans 7 cas (63%), kystique dans 1 cas (9%) et solide dans les 3 cas restants (27%). Les tumeurs souvent volumineuses, leurs tailles variaient de 6 à 35 cm avec une moyenne de 15,8 cm. Les poids variaient de 50 à 3 520 g. Ces tumeurs ont été unilatérales dans 5 cas (45%). Les données des examens d'imagerie en coupe (scanner, imagerie par raisonnance magnétique) n'ont pas été spécifiques de tumeurs non épithéliales de l'ovaire, par contre, elles ont permis dans la majorité des cas d’établir un bilan d'extension et d'opérabilité. La TDM abdominopelvienne a été demandée chez dix patientes. Une seule patiente a bénéficié d'une IRM abdominopelvienne. Les tumeurs germinales ont été représentées par 6 cas avec un cas de dysgerminome pur (Figure 1), trois cas de tératome immature (Figure 2), un cas de tumeur germinale mixte (Figure 3) dont une tumeur du sac vitellin avec composante de dysgerminome et un cas de choriocarcinome non gestationnel, les tumeurs du stroma et du cordon sexuel ont été représentées par 4 cas de tumeur de la granulosa adulte (Figure 4, Figure 5). Nous avons colligé un seul cas de lymphome ovarien primitif type Burkit (Figure 6) (Tableau 4).

**Tableau 3:** Différents aspects échographiques

Description échographique de la masse	Nombre de cas	pourcentage (%)
Kystique multiloculaire	1	(9%)
Kystique uniloculaire	0	
Solidokystique	7	(63%)
Pourcentage liquide/solide faible		7%
Solide	3	(27%)
Necrose dans zones solides	2	

**Tableau 4:** Répartition en fonction du type histologique

Type histologique	Nombre de cas	pourcentage
Tumeur germinales	6	(54%)
Tumeur stroma et cordon sexuel	4	(36%)
Tumeurs autres: lymphome ovarien primitif	1	(9%)

## Discussion

Le cancer de l'ovaire représente 3,7% des cancers de la femme (6^ème^ rang) [1]. Les tumeurs germinales malignes sont excessivement rares représentant environ 5% de l'ensemble des cancers de l'ovaire. Ces tumeurs se rencontrent à tout âge mais sont découvertes le plus fréquemment entre les 1^ère^ et 6^ème^ décennies. Chez l'enfant et l'adolescent, plus de 60% des tumeurs sont d'origine germinale et un tiers d'entre elles sont malignes [2]. Les tumeurs du mésenchyme et du cordon sexuel représentent 6% des tumeurs de l'ovaire. Les tumeurs de la granulosa (TG) sont des néoplasies ovariennes rares, elles représentent 0,6 à 3% de l'ensemble des tumeurs de l'ovaire et 5% des tumeurs malignes de celui-ci [3]. En imagerie, Les tumeurs non épithéliales de l'ovaire ont des aspects différents de la plupart des tumeurs épithéliales. En effet, ces dernières sont de nature essentiellement kystique dans leurs variétés bénigne ou borderline, et de nature mixte kystique et tissulaire dans les carcinomes, associées fréquemment à des végétations d'autant plus nombreuses et volumineuses qu'il s'agit de tumeurs malignes. Les caractères morphologiques des tumeurs non épithéliales sont tout à fait différents. Cela est fondamental pour prévenir le gynécologue et le chirurgien en préopératoire d'une intervention le plus souvent limitée chez l'adulte, ou au contraire de la nécessité d'un bilan radiologique d'extension chez l'adolescente ou l'adulte jeune quand une tumeur maligne est suspectée associé bien entendu au dosage de marqueurs appropriés.

### Les tumeurs germinales

#### Les teratomes immatures

La description des tératomes immatures en imagerie est en accord avec leur présentation macroscopique, soit des tumeurs contenant de la graisse et des portions solides présentant en leur sein de nombreuses structures microkystiques de tailles variées [4, 5]. En échographie, le tératome immature prend la forme d'une tumeur ovarienne à prédominance tissulaire, présentant des zones liquidiennes, des calcifications disséminées et quelques plages graisseuses [6, 7]. Lorsqu'une protubérance de Rokitansky est visible, celle-ci apparaît volumineuse (> 5 cm), irrégulière, à angles de raccordement souvent obtus et pouvant contenir quelques calcifications. En TDM et IRM, la graisse est moins omniprésente au sein des tératomes immatures dont le contenu est séreux ou mucineux, exceptionnellement sébacé. Néanmoins, du tissu adipeux est visible dans la majorité des cas au sein des portions tissulaires de la lésion, correspondant généralement au contingent mature de la tumeur [6, 7].

#### Le choriocarcinome ovarien primitive

En échographie, la tumeur est hétérogène [8]. En scanner, une composante hémorragique peut être visualisée et on note surtout une hypervascularisation majeure faite d’énormes vaisseaux capsulaires entourant la tumeur. Sur les clichés tardifs, l'aspect nécrotique central est retrouvé [8].

#### Le carcinome embryonnaire

Cette tumeur exceptionnelle qui n'a à notre connaissance, jamais été rapportée dans la littérature radiologique est similaire au carcinome embryonnaire testiculaire plus fréquent.

#### Tumeurs germinales mixtes

Ces tumeurs sont composées par plus d'une composante germinale et représentent environ 8% de l'ensemble des tumeurs germinales malignes [6]. L'apparence macroscopique de ces tumeurs est dépendante du type d'association lésionnelle présent, mais il s'agit habituellement de tumeurs complexes à prédominance solide [6].

#### Les tumeurs de la granulosa

En échographie, la forme la plus communément rapportée est une tumeur kystique multicloisonnée avec des cloisons vascularisées en Doppler couleur et pulse [9]. Souvent, on visualise un épanchement dans le cul de sac de Douglas. L’étude échographique doit intégrer l'endomètre, qui peut être hyperplasique, voire néoplasique dans le contexte d'hyperestrogénie [10]. En scanner, un aspect variable a été décrit avec des formes kystiques prédominantes à contenu variable, multiloculaires ou uniloculaires à paroi fine ou épaisse ou alors des formes solides [11]. En IRM, les tumeurs de la granulosa ont le plus souvent, en pondération T2, un aspect kystique multiloculaire avec des composantes solides, mais la tumeur peut également présenter une forme solide de façon uniforme avec un signal intermédiaire. En pondération T1, on note la presence des loculi hyperintenses [12, 13].

### Tumeurs à cellules de Sertoli et stromales

En échographie, l'aspect est peu spécifique, soit d'allure solide, soit kystique multicloisonnée. Le Doppler couleur permet parfois de mettre en évidence une hypervascularisation sur de petites tumeurs dans un contexte d'hyperandrogénie [14]. ces tumeurs n'ont pas de projections papillaires et sont fréquemment accompagnées par un épanchement de Douglas [10]. En scanner, l'aspect décrit est variable, soit kystique multiloculaire, soit solide avec un rehaussement homogène inférieur à celui du myomètre adjacent [15]. En IRM, la présentation morphologique est identique avec des formes solides en isosignal T1 et en signal intermédiaire en pondération T2. L'IRM dynamique injectée met en évidence une prise de contraste supérieure aux tumeurs du groupe fibrothécal mais nettement inférieure aux tumeurs épithéliales solides malignes.

### Les tumeurs à cellules steroides

En imagerie, la petite taille habituelle de ces tumeurs explique leur absence éventuelle de détection par échographie et scanner [15]. Le Doppler couleur, l'angioscanner pelvien ou l'IRM dynamique apparaissent utiles pour démasquer l'hypervascularisation majeure de ces tumeurs [16].

### Gynandroblastome

Il s'agit d'une tumeur exceptionnelle associant des composantes cellulaires bien différenciées de type granulosa (ovarienne) et de type Sertoli (testiculaire) mais aucun cas radiologique n'est actuellement rapporté.

## Conclusion

Bien que les tumeurs malignes rares ovariennes soient un groupe de tumeurs hétérogènes, elles partagent, en plus de leur rareté, plusieurs des caractéristiques suivantes par rapport aux tumeurs communes gynécologiques: la survenue à un âge moyen plus précoce, des marqueurs tumoraux propres, une génomique spécifique, une présentation histologique parfois trompeuse et/ou un diagnostic de malignité parfois difficile, un stade plus souvent localisé, une chirurgie dont un objectif majeur est de préserver la fécondité, autant que l’état carcinologique le permet, un pronostic le plus souvent favorable après une prise en charge adéquate.

## References

[1] Ferlay J, Shin HR, Bray F, Forman D, Mathers C, Parkin DM (2010). Estimates of worldwide burden of cancer in 2008: GLOBOCAN 2008. Int J Cancer..

[2] Scully RE, Young RH, Clement PB (1998). Germ cell tumors Tumors of the ovary and maldeveloped gonads, fallopian tube, and broad ligament Washington, Armed Forces Institute of Pathology.

[3] Bompas E, Freyer G, Vitrey D, Trillet-Lenoir V (2000). Tumeur à cellules de la granulosa: revue de la littérature. Bull Cancer..

[4] Yamaoka T, Togashi K, Koyama T, Fujiwara T, Higuchi T, Iwasa Y (2003). Immature teratoma of the ovary: correlation of MR imaging and pathologic findings Eur. Radiol..

[5] Bazot M, Cortez A, Sananes S, Boudghene F, Uzan S, Bigot JM (1999). Imaging of dermoid cysts with foci of immature tissue. J Comput Assist Tomogr..

[6] Brammer HM, Buck JL, Hayes WS, Sheth S, Tavassoli FA (1990). From the archives of the AFIP, malignant germ cell tumors of the ovary:radiologic-pathologic correlation. radiographics..

[7] Bazot M, Crtez A, Sananes Suzan, SBigot JM (1999). Imaging of dermoid cystswith foci of immature tissue. J Computassist tomogr..

[8] Bazot M, Cortez A, Sananes S, Buy JN (2004). Imaging of pure primary ovarian choriocarcinoma. Am J Roentgenol..

[9] Hong BK, Jeng CJ, Huang SH, Yang YC, Wang KG (1996). Sonographic and clinical findings of granulosa cell tumor Chung Hua. I Hsueh Tsa Chih..

[10] Bats A-S, Larousserie F, Le Frère-Belda MA, Metzger U, Lécuru F (2009). Tumeurs non épithéliales malignes de l'ovaire. Gynécologie Obstétrique & Fertilité..

[11] Ko SF, Wan YL, Ng SH, Lee TY, Lin JW, Chen WJ (1999). Adult ovarian granulosa cell tumors: spectrum of sonographic and CT findings with pathologic correlation. Am J Roentgenol..

[12] Kitamura Y, Kanegawa K, Muraji T, Sugimura K (2000). MR imaging of juvenile granulosa cell tumour of the ovary: a case report Pediatr. Radiol..

[13] Tanaka YO, Tsunoda H, Kitagawa Y, Ueno T, Yoshikawa H, Saida Y (2004). Functioning ovarian tumors: direct and indirect findings at MR imaging. Radiographics..

[14] Yanushpolsky EH, Brown DL, Smith BL (1995). Localization of small ovarian Sertoli-Leydig cell tumors by transvaginal sonography with color Doppler Ultrasound Obstet. Gynecol..

[15] Outwater EK, Wagner BJ, Mannion C, McLarney JK, Kim B (1998). Sex cord-stromal and steroid cell tumors of the ovary. Radiographics..

[16] Wang PH, Chao HT, Lee RC, Lai CR, Lee WL, Kwok CF (1998). Steroid cell tumors of the ovary: clinical, ultrasonic, and MRI diagnosis--a case report. Eur J Radiol..

